# New Parameter for Benchmarking Plasmonic Gas Sensors Demonstrated with Densely Packed Au Nanoparticle Layers

**DOI:** 10.1021/acsami.4c11102

**Published:** 2024-10-14

**Authors:** Manuela Proença, Tomáš Lednický, Diana I. Meira, Marco S. Rodrigues, Filipe Vaz, Joel Borges, Attila Bonyár

**Affiliations:** † Physics Center of Minho and Porto Universities (CF-UM-UP), University of Minho, Campus de Azurém, 4800-058 Guimarães, Portugal; ‡ Leibniz Institute of Photonic Technology, Albert-Einstein-Str. 9, 07745 Jena, Germany; § LaPMET - Laboratory of Physics for Materials and Emergent Technologies, University of Minho, Campus de Gualtar, 4710-057 Braga, Portugal; ∥ Department of Electronics Technology, Faculty of Electrical Engineering and Informatics, Budapest University of Technology and Economics, Egry József street 18, H-1111 Budapest, Hungary; ⊥ Wigner Research Centre for Physics, Konkoly-Thege Miklós way 29-33, H-1121 Budapest, Hungary

**Keywords:** well-ordered gold nanoparticles, localized surface plasmon resonance, refractive index gas sensing, modeling adsorption interfaces, gas sensitivity benchmarking parameter

## Abstract

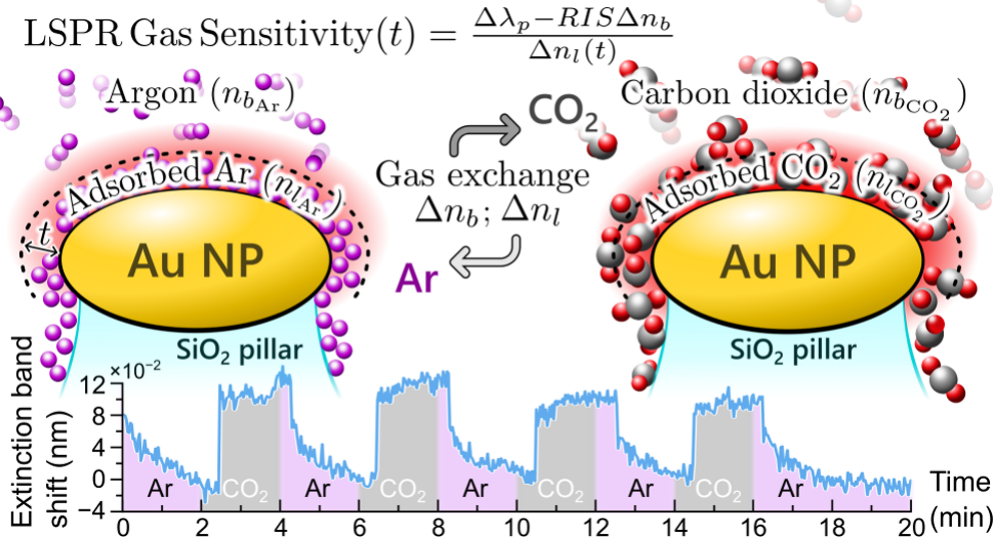

Localized surface plasmon resonance (LSPR) gas sensitivity is introduced as a new parameter to evaluate the performance of plasmonic gas sensors. A model is proposed to consider the plasmonic sensors’ surface sensitivity and plasmon decay length and correlate the LSPR response, measured upon gas exchange, with an equivalent refractive index change consistent with adsorbed gas layers. To demonstrate the applicability of this new parameter, ellipsoidal gold nanoparticles (NPs) arranged in densely packed hexagonal lattices were fabricated. The main advantages of these sensors are the small and tunable interparticle gaps (18–29 nm) between nanoparticles (diameters: 72–88 nm), with their robust and scalable fabrication technology that allows the well-ordered arrangement to be maintained on a large (cm^2^ range) area. The LSPR response of the sensors was tested using an LSPR sensing system by switching the gas atmosphere between inorganic gases, namely He/Ar and Ar/CO_2_, at constant pressure and room temperature. It was shown that this newly proposed parameter can be generally used for benchmarking plasmonic gas sensors and is independent of the type and pressure of the tested gases for a sensor structure. Furthermore, it resolves the apparent disagreement when comparing the response of plasmonic sensors tested in liquids and gases.

## 1 Introduction

The air we breathe contains many harmful gases and compounds from different sources, which can threaten human health. The use of sensors is therefore essential for monitoring gas levels in the environment, but also for a wide range of other applications, including medical diagnostics, leak detection, poisonous gas emissions in industries, food control, and agriculture.^1−4^ For example, precise measurement of carbon dioxide (CO_2_) concentration in the atmosphere is of paramount importance for studying the cascading effects of global warming.^5,6^ Most currently prevalent gas sensors are based on electrical conductivity changes and are usually made up of metal oxides, owing to their outstanding sensing response to various target gases and good chemical stability.^5−8^ However, the high operating temperature and cross-sensitivity to other gases restrict the application of this detection technology. Therefore, many continuing efforts are in progress to develop new techniques for the realization of gas sensors operating at room temperature with high sensitivity and selectivity toward different analytes.^9−11^

Among some alternatives, optical-based gas sensors have gained enormous relevance over the past few years.^12,13^ In particular, sensors based on the localized surface plasmon resonance (LSPR) phenomenon.^14−17^ have emerged as a promising alternative to the conductometric gas sensors due to enhanced sensitivity, the possibility of being selective and specific, and other characteristics such as miniaturization, fast response, and room temperature operation.^18−20^

The LSPR phenomenon is spatially confined to noble metal nanoparticles (NPs) or nanostructures. It occurs when the incident light frequency coincides with the oscillation frequency of the conduction band electrons of a nanoparticle, causing strong light extinction (absorption and scattering) at specific resonant wavelengths. Excitation of localized surface plasmons on metallic nanoparticles results in local electromagnetic field enhancement, dramatically affecting molecules adsorbed in their close vicinity.^21,22^ Therefore, LSPR-based sensors may be capable of distinguishing extremely small changes in the refractive indexes of the surrounding media;^15,23^ to monitor biomolecular events^24−26^ and the adsorption of molecules;^27−29^ or even to detect charge transfer events caused by self-assembled monolayers.^30^ These events can be quantified through shifts and modifications of the LSPR band.^31^ Yet, contrary to biomolecular detection, high-sensitivity gas sensing at room temperature is significantly more challenging because the refractive indexes of gas molecules closely approximate air. Various research groups have successfully detected volatile organic compounds (VOCs).^32−35^ However, LSPR sensing based on bulk RI changes has a clear difficulty detecting inorganic gases, such as CO_2_, SO_2_, H_2_, or N_2_, at room temperature, and only a limited number of works have been reported in this field.^36−40^ Unlike most VOCs, inorganic gases exist under ambient conditions, and their refractive indexes are only different from the air by approximately 10^–4^ refractive index units (RIU). Therefore, using high-resolution (HR) LSPR spectroscopy systems is paramount in such cases, such as the one developed by Bingham et al.,^38^ which has proven effective in resolving shifts in the order of Δλ = 10^–2^ nm. The same group (Van Duyne group)^39^ also detected CO_2_ concentrations higher than 10% in N_2_ by coating a triangular Ag nanoparticle array with a metal–organic framework as an absorbent. More recently, Proença et al.^40^ developed a homemade HR-LSPR sensing system, able to distinguish LSPR signals from different tested inert gases using a nanocomposite-based sensor.

Currently, many nanostructures such as noble metal nanoparticles (e.g., gold, silver) are designed for different LSPR sensing fields,^41−45^ including gas sensing.^40,46^ Increasing the gas sensitivity while simplifying the sensor structure is still one of the most dynamic research issues. LSPR-based sensors’ sensing performance depends on several parameters of the plasmonic NPs, including the size, shape, and composition (e.g., bimetallic or core–shell structures).^47,48^ In particular, by arranging plasmonic multi-NPs into a defined geometry, interparticle coupling effects may occur and can significantly reduce the line width (full width at half-maximum) of the plasmonic resonance.^49,50^ Furthermore, decreasing the interparticle gap and, consequently, increasing the coupling between NPs, leads to a sensitivity improvement of the sensor.^51^ However, the sensor’s selectivity is still a significant drawback, which can only be improved through surface functionalization to achieve the necessary chemoselectivity.^52,53^

The sensing performance of LSPR-based sensors is generally evaluated by their bulk RI sensitivity (RIS), defined as the LSPR band shift (Δλ) per RI difference (Δ*n*).^54^ RIS is also widely used to benchmark the performance of plasmonic sensors, where the bulk refractive index difference of the measured gases is used for the calculation.^55−57^ The problem with this approach is that the resulting RIS values calculated this way are usually 1 order of magnitude higher than those calculated by calibrating the same LSPR sensor with liquids of known refractive indexes. Hence, based on the literature review, one can claim that there is a clear disagreement between calibration methods in liquids and gases.

To address this issue and provide proper means to assess the gas sensing performance of plasmonic structures, a new parameter termed “LSPR gas sensitivity” is introduced in this work. A new model is proposed, which considers adsorbed gas layers and the plasmon decay length of the structures to interpret the LSPR peak shift signal experienced upon changing gases. To demonstrate the applicability of this new benchmarking parameter, hexagonally ordered Au NP layers (quasi-arrays) were fabricated to be used for the gas sensing tests as a case study. This type of ordered arrangement offers tailored nanostructures, where the nanoparticle size and interparticle gap can be tuned in a wide range (72–88 nm diameter and 18–29 nm gap size). Tunable plasmonic coupling and resonance peak position are beneficial in many sensing areas,^58,59^ and the effect of varying interparticle gaps on the gas sensing performance has been demonstrated in literature.^60^

The plasmonic sensors were fabricated by combining electrochemical and thin-film technologies, including porous anodic alumina (PAA) anodizing, thin-film deposition, template-assisted solid-state dewetting, and reactive ion etching. The cost-effectiveness of this fabrication method is due to its ability to provide appropriate homogeneity over a large surface area (several cm^2^).^61^ Three sensor types were thoroughly characterized regarding their nanomorphology, and relevant Au NPs distribution parameters. Their plasmonic properties were studied in terms of bulk refractive index sensitivity, in response to liquid environments, and regarding plasmonic gas sensitivity. The obtained results point out the discrepancies between the sensitivities measured in liquids and gases, revealing the pertinence of introducing this new model for the proper interpretation of gas sensing response.

## 2 Experimental Section

### 2.1 Fabrication of Au NP Layers (Single, Double, Triple)

**Figure 1:**
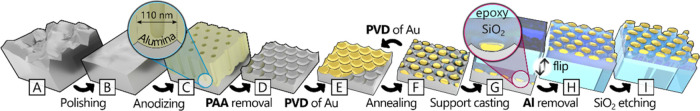
Schematic illustration of the fabrication process of the Au NP layers. (A–D) Synthesis of aluminum nanostructured template through porous anodization and selective PAA removal. (D–F) Formation of Au NP layer by solid-state dewetting of a thin Au film over the Al template. (F–I) Transfer of the Au NP layer to a transparent SiO_2_ substrate and its controlled etching to achieve properly exposed Au NPs on SiO_2_ nanopillars.

**Figure 2:**
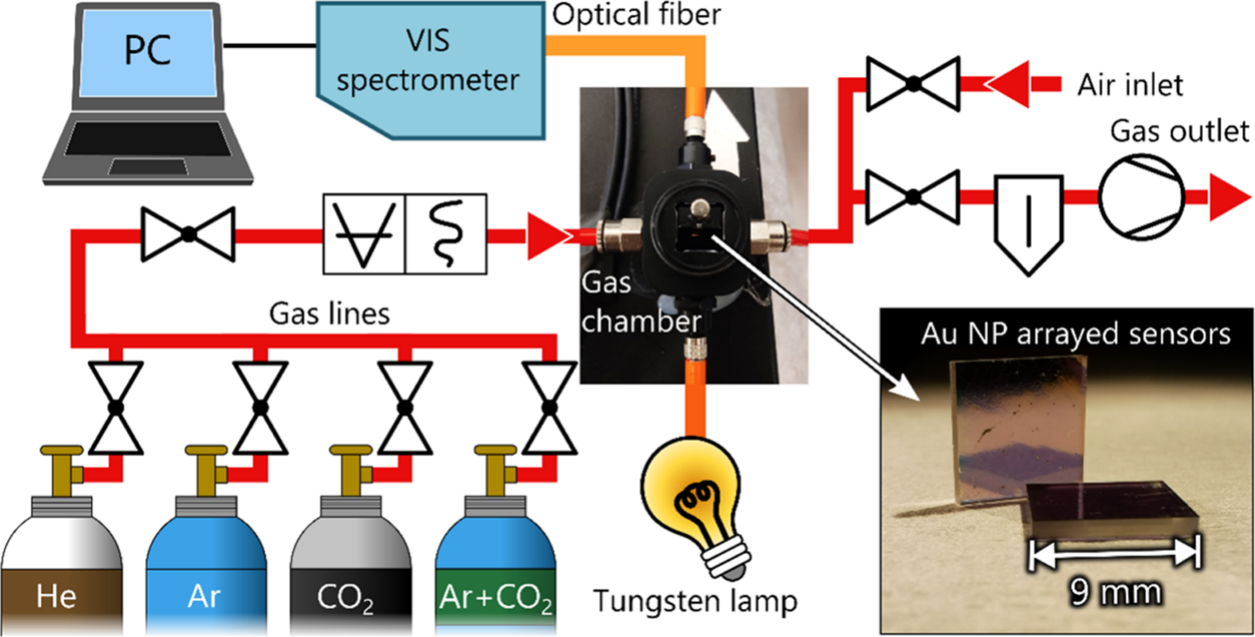
Schematic diagram of the HR-LSPR spectroscopy system, composed of a gas chamber connected to gas controls, a vacuum unit, and gas cylinders. The gas chamber is equipped with optical and gas in/outputs and a holder for the plasmonic sensor.

The fabrication technology (illustrated in Figure 1) of Au NP layered sensors is based on a previous work that presents detailed optimization of the technological parameters.^61^ Here, Au NP layers were prepared in the same way over aluminum templates with ∼110 nm cell size and in the three size distributions (type B1–3).^61^ In summary, a 250 μm thick high-purity (99.999%) aluminum sheet (Goodfellow) was cut into 25 × 50 mm^2^ pieces, cleaned, annealed (550 °C, ∼10^–4^ Pa, 15 h), followed by mechanical and electrochemical polishing (Figure 1A,B). Afterward, sheets were anodized (40 V, 0.3 M oxalic acid, 8 °C, 20 h) to form a well-ordered porous anodic alumina (PAA) over the surface that was selectively removed, resulting in a nanobowled Al template (Figure 1C,D). The Au NP layers were synthesized utilizing a template-assisted solid-state dewetting of the magnetron-sputtered Au film (Figure 1D–F). Three different size distributions were achieved by repeating this process of thin film deposition (0.35 × 10^–1^ nm s^–1^, 10^–1^ Pa): single (8 nm), double (8 + 6.5 nm), and triple (8 + 6.5 + 5 nm); and annealing on a hot plate (300 °C for 1 min). After synthesis, Au NP layers were plasma cleaned for 5 min (300 W, 20:80% of Ar:O_2_ atmosphere, 50 Pa, Diener Tetra 30). This plasma treatment occurred prior to a thin 20 nm SiO_2_ layer deposition. The SiO_2_ layer was deposited by e-beam evaporation (to avoid PECVD chamber contamination by gold) and then increased to ∼300 nm by PECVD (40 min, ICP = 600 W, 400 Pa, 3:13 sccm of SiH_4_:N_2_O, Oxford Instruments Plasma Technology PlasmaPro 100). The PECVD process was chosen to avoid high stress and cracking of the SiO_2_ layer. The formed SiO_2_/Au NPs/Al sandwich structure was then glued by a two-compound epoxy (Loctite 3430) to a 1.1 mm thick glass slide (Figure 1E–G). Then, the backside of the Al sheet was gently scratched with sandpaper to damage the oxide layer and promote the etching speed of aluminum in an HCl (35% w/w) and CuCl_2_ (2 M) water solution (Figure 1G,H). Samples were thoroughly rinsed in distilled water and dried by an air stream. Transferred Au NP layers are still embedded in the SiO_2_ substrate (Figure 1H), which hinders their accessibility and, thus, the performance of the LSPR sensor. Therefore, SiO_2_ substrate was partly etched (2 min, 100 W, 4 Pa, 30:15:5 sccm of Ar:CHF_3_:O_2_) with short precleaning (1 min, 100 W, 13.3 Pa, 20 sccm O_2_) by RIE (Oxford Instruments Plasma Technology PlasmaPro 80 RIE) to mostly reveal embedded Au NPs, forming column-like structure (Figure 1I), aiming to increase its sensitivity. Lastly, the glass samples with Au NP layers of 25 × 50 mm^2^ active area were cut to 9 × 9 mm^2^ sensors (photograph in Figure 2) by a dicing saw, washed in isopropyl alcohol, and dried by nitrogen stream.

### 2.2 Morphological Characterization of the Au NP Layers

The morphology of the Au NP layers was studied by a high-resolution SEM ThermoFisher Verios 460 L in the immersion mode with a through-the-lens detector with the charge-neutralization mode at 5 keV and 25 pA. Scanning transmission electron microscopy (STEM) was performed with a dual-beam SEM/FIB system (FEI Helios NanoLab 660) using a STEM detector in bright-field (BF) mode and an operating voltage of 23 keV. Samples were prepared using a thin carbon layer (15–20 nm) by flashing carbon thread deposition (Leica ACE600) as a conductive layer and using FIB-deposited carbon as a capping layer. The images presented were postprocessed (e.g., contrast, charging artifacts) in Gwyddion software (v. 2.60). The Au NP layer distributions’ analysis was performed over SEM micrographs (5 × 3.3 μm^2^, ∼1500 NPs) by a MATLAB algorithm.^40^ For more details on the calculation of NP parameters, and corresponding values, please see Supporting Information S1 (Table S1).

### 2.3 Refractive Index Sensitivity and Gas Sensing Tests Using HR-LSPR Spectroscopy

The sensing properties of the Au NP layers (single, double and triple types) were first evaluated by estimating their bulk refractive index sensitivity (RIS). RIS is calculated by LSPR band shifts induced by a change in the refractive index of the (liquid) environment. The transmittance spectra of the sensors were monitored in real-time in a custom-made high-resolution LSPR spectroscopy system (Figure 2), described in more detail elsewhere.^40^ Prior to optical measurements, the surface of the Au NP layers was cleaned with O_2_ plasma (1 min, 30 W, 80 Pa) to remove hydrocarbon contamination, as previously verified.^61^ For measurements in liquids, the sensors were immersed into aqueous sucrose solutions: 0, 10, 30, and 50% (w/w) (*n* = 1.333, 1.3478, 1.3812, and 1.4201 RIU). Each spectrum was acquired by a modular spectrometer (Ocean Optics HR4000 model) using an average of 500 scans of 4 ms integration time. The repeatability of the sensing test was assessed in 5 cycles of 1 min monitorization, spaced between cleaning practices, in each Au NP layer sample. The data was processed by the NANOPTICS software,^62^ that allows to rapidly identify and quantify changes and shifts in the LSPR band. Finally, the bulk RIS value was calculated from the slope of the linear fit from the plot of wavelength at transmittance (LSPR) band minimum (extinction maximum), as a function of the refractive index.

Gas sensitivity tests were performed in the described system (Figure 2), with an active vacuum unit to maintain a constant gas pressure inside the chamber. The base pressure of the system is 2 × 10^2^ Pa. The gas sensing performance of the three distinct Au NP layers was obtained using atmospheres of He (99.995% purity) and Ar (99.995% purity), switching the gases every 120 s until at a working pressure of 2 × 10^4^ Pa. The LSPR band, observed in the transmittance spectra, was monitored in real-time. He/Ar gas cycles were performed to ensure the reproducibility of the sensing procedure while all experiments were carried out at room temperature. To smooth the spectra and to find the wavelength peak position (minimum transmittance peak at the LSPR band) over time, the software NANOPTICS was used, which also determined the average wavelength shifts of the LSPR spectra caused by the exposure to different gaseous atmospheres, and the signal-to-noise ratio (SNR) of each gas sensitivity test. Then, additional measurements were performed using Ar as background gas against CO_2_ and a mixture of 20% of CO_2_ in Ar for the best-performing sensor. The refractive indexes ((*n* – 1) × 10^6^) of 8.75 (He), 70.25 (Ar), 78.65 (20% of CO_2_ in Ar), and 112.25 (CO_2_) were used for calculations, scaled linearly with pressure according to refs.^63,64^

## 3 Results and Discussion

### 3.1 Characterization of the Au NP Layers/Au NP Arrangement Control

**Figure 3:**
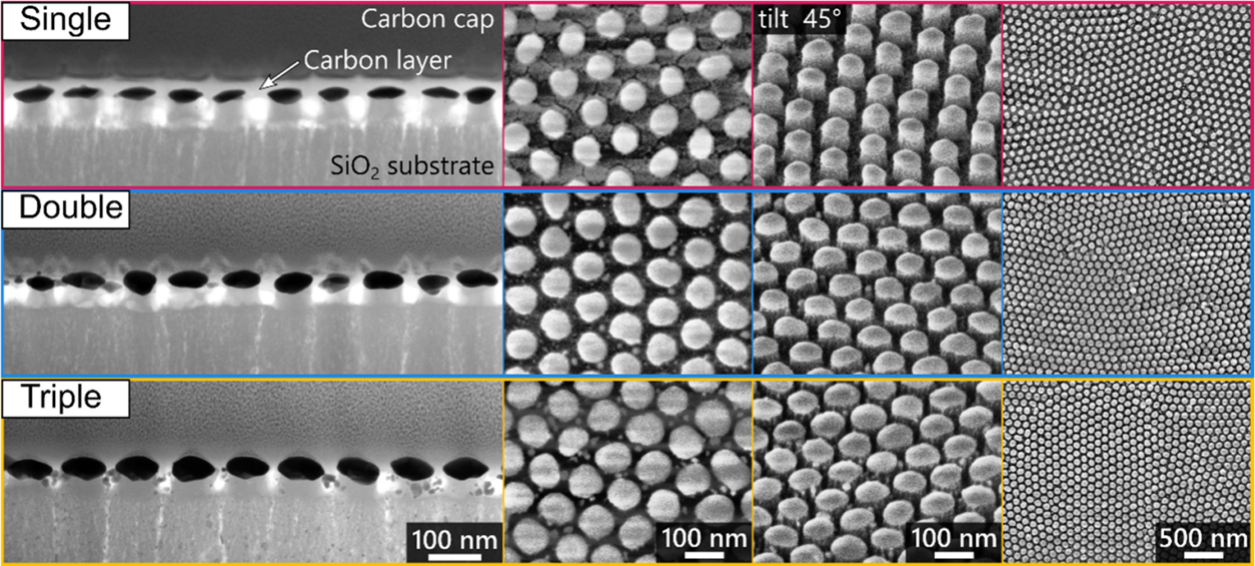
STEM (cross-view) and SEM images of three types of Au NP arrangements on SiO_2_ nanopillars.

The morphology and arrangement of three different types of Au NP layers are shown in Figure 3, with more details in Supporting Information S1 (Figures S1 and S2). Au NPs on SiO_2_ pillars exhibit an oblate spheroid shape, with the diameter and thickness increasing in the following order: single, double, and triple arrangements (see Table 1). The dewetting process on the Al template (Figure 1F) and subsequent flipping of Au NPs (Figure 1H) results in a smooth top surface, while the bottom surface retains its structured nature, characterized by facets and twinning sites. The close-range arrangement of the Au NPs is consistent across all types, as it is derived from the same fabrication step (Figure 1D). It is worth noting that, as discussed in previous works,^61,65^ an increase in the size of Au NPs leads to a higher number of defects, including small satellite particles and merged adjacent Au NPs.

**Table 1:** Distribution Parameters of Au NPs Arrangements

Au NP qarrangement	Au NP diameter (nm)^a^	Au NP height (nm)^b^	nearest neighbor distance (nm)^c^
single	73 ± 4	29 ± 5	38 ± 10
double	82 ± 3	33 ± 6	23 ± 6
triple	88 ± 4	41 ± 5	16 ± 6

a Set of: ∼100 NPs.

b >20 NPs.

c >250 distances.

**Figure 4:**
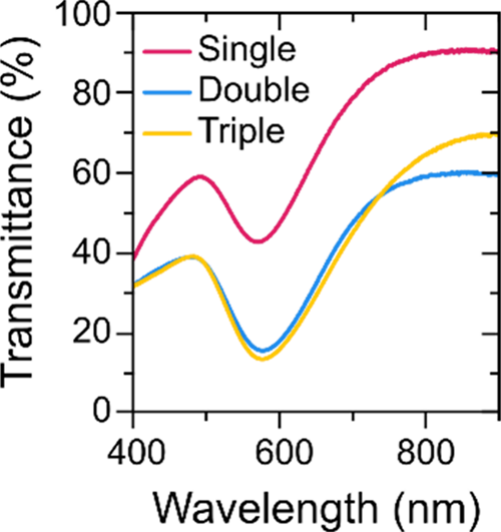
LSPR bands in transmittance mode for single (pink), double (blue) and triple (yellow) Au NP arrangements.

The morphology acquired by the different Au NP arrangements is expected to influence their plasmonic (LSPR band) response and, hence, their sensing performance. The transmittance spectra (T %) of the Au NP layers show transmittance minima due to LSPR behavior that corresponds to maximum extinction, mainly due to light scattering. The T-LSPR bands for the three configurations are shown in Figure 4. All transmittance bands are characterized by a transmittance minimum for a wavelength very close to 570 nm. A measurable decrease of the transmittance band is observed, particularly from single Au NP layers to double and triple layers. The increase in the NP size from single to double types augments the scattering efficiency, especially because the height distribution is higher (i.e., higher layer thickness), which explains the overall decrease in the transmittance spectra. The same argument cannot be used to explain the rather similar spectra between double and triple types. Even though the diameter did not change as much as from single to double, considering the associated uncertainties, the height of Au NPs continued to increase. Nevertheless, as aforementioned, the triple arrangement that produces higher Au NP sizes, is more prone to yield structural defects, and this might explain such unexpected optical behavior. Anyway, the T-LSPR band is well-defined, though not very different from the double arrangement.

### 3.2 Bulk RIS Determination for the Au NP Layers

**Figure 5:**
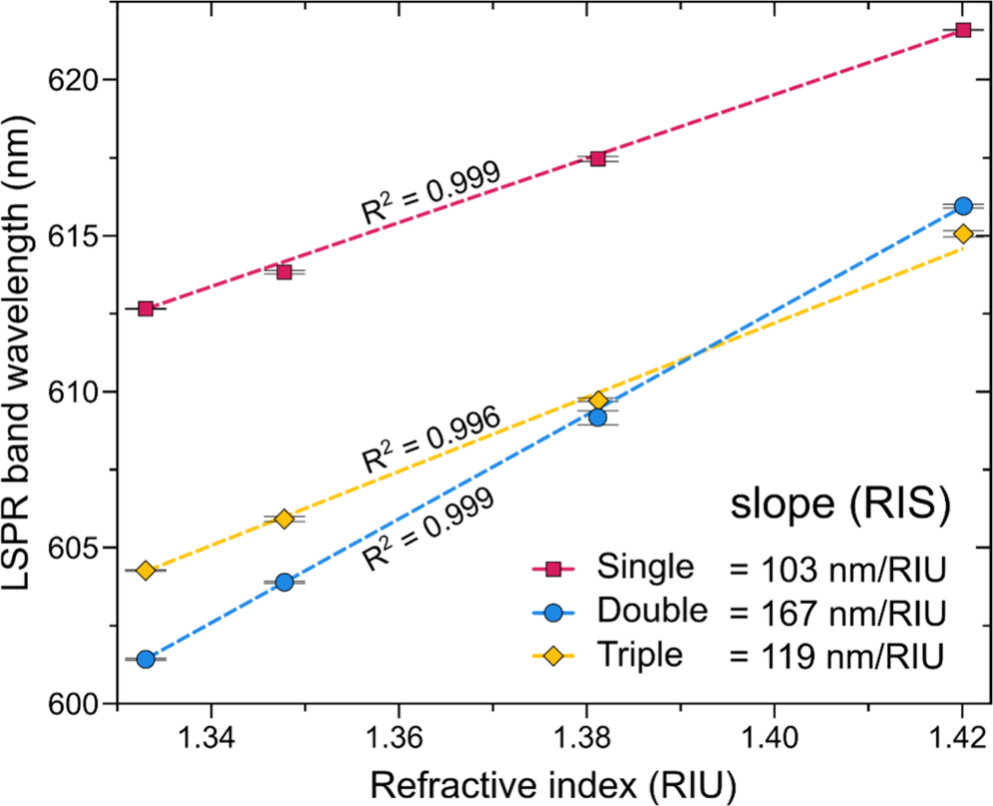
LSPR band wavelength shift of the single (pink), double (blue), and triple (yellow) Au NP layers as a function of the RI of the surrounding liquids (aqueous sucrose solutions). The solid line is the linear fit to the data and the slope corresponds to the RIS value.

The Au NP layers sensing performance was accessed by the ability to discriminate various refractive indexes (liquid environment) of the surrounding medium through optical response modifications, namely T-LSPR band shifts (Δλ_p_), as depicted in Figure 5. As evidenced, the wavelength at the transmittance minimum of the Au NP layers suffered a redshift as the RI increased, within a range from 1.333 to 1.420 RIU, allowing RIS determination through the slope of the linear fit. The sensitivity of the different Au NP arrangements in liquids was estimated to be above a hundred, detailed as 103 ± 1 (nm RIU^–1^), 167 ± 1 (nm RIU^–1^), and 119 ± 4 (nm RIU^–1^) for the single, double and triple Au NP layers, respectively. It is thus evident that the different Au NP arrangements influenced the sensing ability, as in fact already demonstrated for similar Au NP layer arrangements reported in a previous work.^61^

The increase in the NP size (diameter and height) augments the scattering efficiency, while the reduction of the nearest neighbor distance enhances plasmon coupling. This leads to an increase in the near-field intensity between the gap, which causes a sensitivity increase from single to double arrangements. Regarding the triple arrangement, once again, the existence of more structural defects during fabrication, results in a plasmonic behavior where the double Au NP layer shows better sensing results than the triple.

### 3.3 Gas Sensitivity Tests

#### 3.3.1 Inert Gas Responses of the Au NP Layers

**Figure 6:**
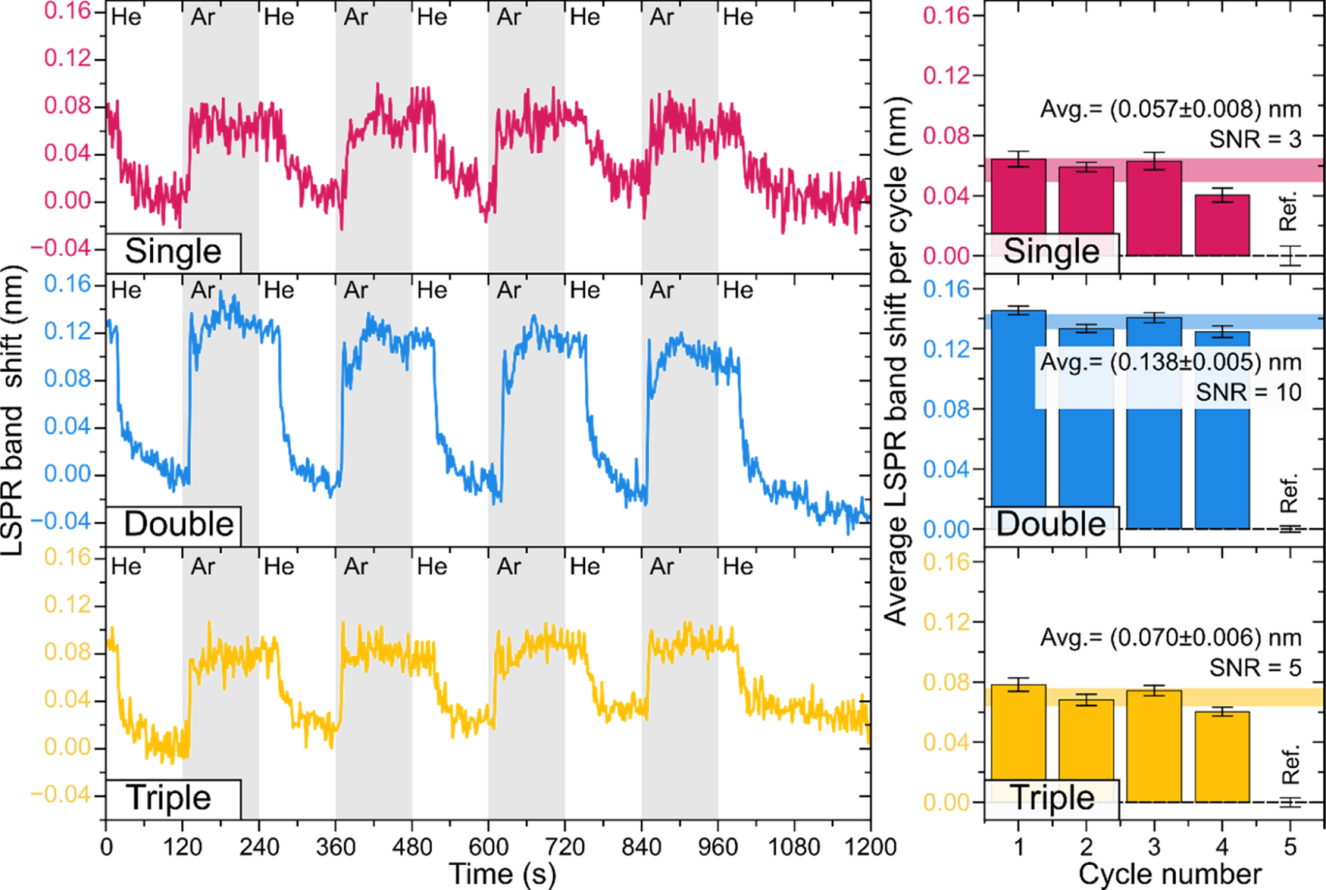
LSPR band wavelength shift measurements of single (pink), double (blue), and triple (yellow) Au NP layers during the He vs Ar cycles (left). The graphic representation of the average wavelength shift for each cycle is depicted to the right.

The LSPR sensing response, measured as a wavelength shift, Δλ_p_, of the plasmonic Au NP layers was evaluated by switching inert He and Ar gases in a HR-LSPR spectroscopy system. The tests were performed at room temperature and at a constant pressure of 2 × 10^4^ Pa. The typical test involves 5 cycles, where the last one is used for reference by using two half-cycles of the background gas (He), and then the data is processed by the software.^62^ Real-time LSPR responses to Ar gas for single, double, and triple NP layers are displayed in Figure 6, with perfectly noticeable cyclic band shifts between each gas (He and Ar). In all the arrangements, the LSPR peak shifts to longer wavelengths during Ar exposure and then returns to the initial position after reintroducing the He gas. This behavior is due to the higher RI of Ar relative to He. The average LSPR wavelength shifts and signal-to-noise ratio (SNR) values were estimated Δλ_p_ = 0.057 ± 0.008 nm (SNR = 3), Δλ_p_ = 0.138 ± 0.005 nm (SNR = 10), and Δλ_p_ = 0.070 ± 0.006 nm (SNR = 5) for single, double, and triple NP layers, respectively. Hence, as observed by the bulk RIS values, the double NP layer revealed the best performance, possessing the highest LSPR response to target gas, which is almost a 2-fold increase compared to the triple NP layer and 2.5 times higher than the single arrangement. Furthermore, long-term stability studies performed on the double Au NP layer showed a small decrease in LSPR response in the investigated in a six-month period (Supporting Information S2 and Figure S3).

Although all samples showed outstanding LSPR responses when compared with, e.g., nanocomposite Au/CuO thin films tested under the same conditions,^40^ the double NP layer proved to be the most promising arrangement to be further examined as an LSPR-based gas sensor, in agreement with sensing tests with liquid environments (Section 3.2).

#### 3.3.2 LSPR Response of the Double Au NP Layer to CO_2_ Gas

**Figure 7:**
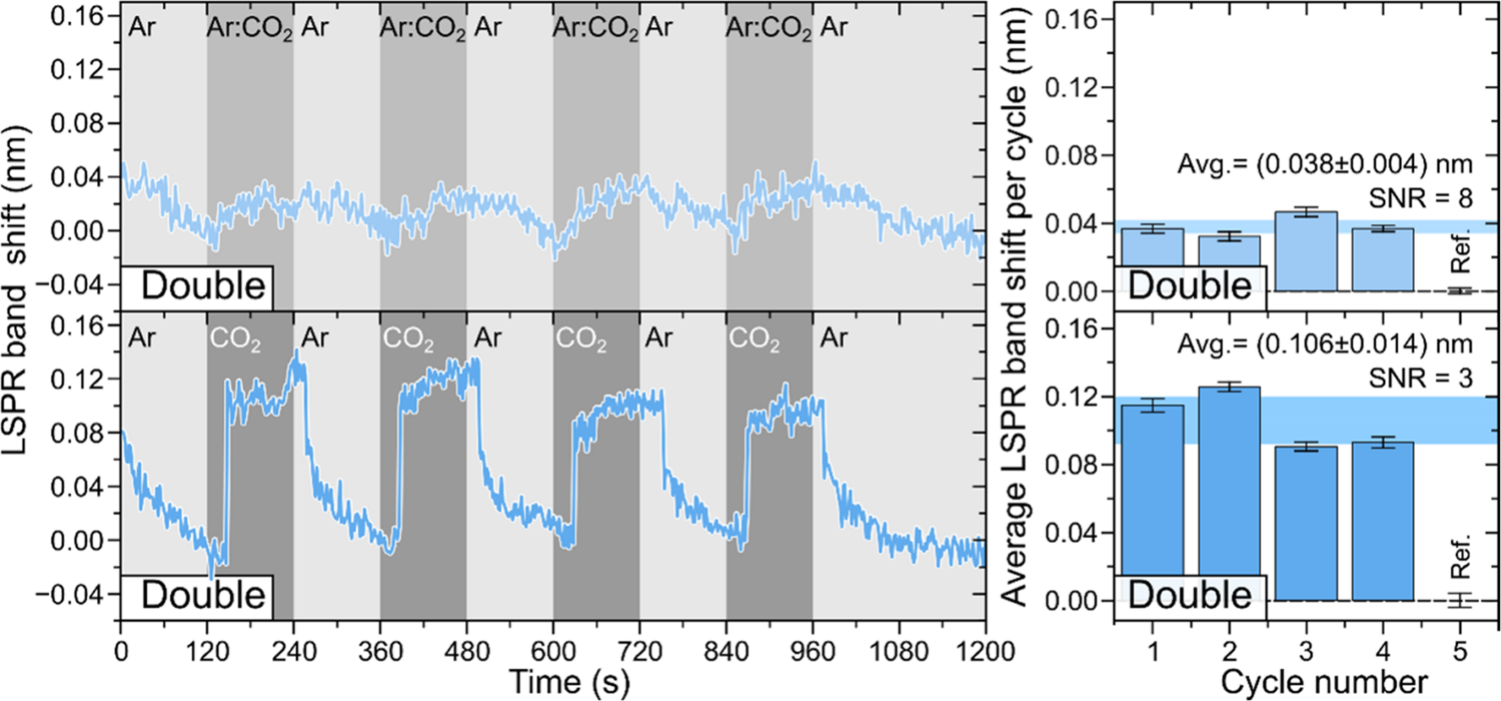
LSPR band wavelength shift monitoring of double Au NP layers for Ar/Ar:CO_2_ and Ar/CO_2_ gas cycles over time (left). The graphic representation of the average wavelength shift for each cycle is depicted to the right.

To further corroborate the hypothesis that the double Au NP layer performs well as an LSPR-based gas sensor, sensing tests were accomplished using CO_2_ gas and a mixture of 20% of CO_2_ in Ar (Ar:CO_2_), using Ar gas as background gas. Similarly to previous tests, the gaseous atmosphere inside the HR-LSPR spectroscopy system was switched for 5 cycles, at room temperature, between either Ar and Ar:CO_2_ or pure CO_2_ (Figure 7). As expected, the LSPR wavelength peak red-shifted when the gaseous atmosphere changed from Ar to Ar:CO_2_ or pure CO_2_ due to the higher RI of CO_2_ than Ar. Moreover, the arrangement response changed as a function of CO_2_ concentration. The average peak shift amount was Δλ_p_ = 0.038 ± 0.004 nm (SNR = 3) after the introduction of Ar:CO_2_ gas and Δλ_p_ = 0.106 ± 0.014 nm (SNR = 8) after the introduction of CO_2_ gas.

Using the IUPAC definition,^66^ the LOD value of the double Au NP layer was estimated to be 4.4 × 10^–6^ RIU, corresponding to a detection limit as low as 10% for CO_2_. This detection limit for CO_2_ detection is close to the results reported by the Van Duyne group,^39^ yet using triangular Ag nanoparticle arrays to build the LSPR sensor. More recently, Pohl et al.^67^ reported the detection of CO_2_ at ppm level, however the measurements were not performed at room temperature.

These results demonstrate that the double Au NP layer introduces a superb sensing potential to be applied as a transducer matrix in developing an LSPR-based gas sensor or even in other types of chemosensors.

### 3.4 Interpretation of the Gas Sensing Response

In the previous two sections, the response of Au NP layers in the presence of different gases was demonstrated. In order to evaluate the sensing performance, the first thing that needs to be noted is that bulk RI changes alone cannot account for the measured signals. If RIS values of the LSPR-based sensors are calculated as defined in eq 1, considering the bulk RI of the different gases (or gas mixture), the result would be an order of magnitude higher than what was calculated with liquids (Figure 5).
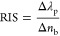
1

For example, for the double Au NP arrangement, between He and Ar gases (Figure 6) the measured peak shift is Δλ_p_ = 0.138 nm, and the difference in bulk refractive indexes is Δ*n*_b_ = 0.0000615, which would result in a RIS of 2243 nm RIU^–1^, much higher than the 167 nm RIU^–1^ estimated for liquid environment (Figure 5). Although this approach is clearly controversial, it is used in the literature for benchmarking the performance of gas sensors.^55−57^

**Figure 8:**
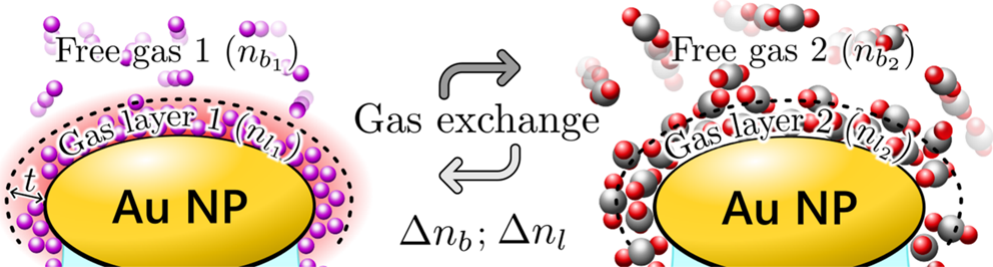
Illustration of the gas exchange process on the top of the ellipsoidal gold nanoparticles. Nanoparticle and gas molecule sizes are not to scale in this model.

To resolve these apparent discrepancies between sensitivity calibrations in liquids and gases, and for the proper evaluation of the sensors’ response upon gas sensing, a gas layer formed on the surface of the nanoparticles should be considered. This gas layer can be modeled with an effective thickness (*t*) and refractive index (*n*_l_). Unlike the bulk RI of the free gases (*n*_b_), the exact values of *t* and *n*_l_ are not known and are expected to vary significantly between the different gases. Depending on the pressure, temperature, and surface properties, the different types of gas atoms/molecules can form gas mono- or multilayers,^68^ with different density and hence refractive index. For this reason, one can conveniently model the response of the sensors with coupled (Δ*n*_l_ – *t*) pairs, or functions, where Δ*n*_l_ represents the RI change in a layer with an arbitrarily selected thickness, *t*. The gas exchange process is illustrated in Figure 8, where Δ*n*_l_ = *n*_l_2__ – *n*_l_1__ and Δ*n*_b_ = *n*_b_2__ – *n*_b_1__.

The peak shift of a plasmonic sensor (Δλ_p_) caused by forming a gas layer on the (initially clean) nanoparticles’ surface can be expressed by eq 2(69) (assuming an exponential plasmon decay length), where *n*_b_ is the bulk refractive index of the gas, which forms the layer, ξ_D_ is the plasmon decay length for the nanoparticle arrangement, and RIS is the refractive index sensitivity as previously defined in eq 1 (sometimes also referred to as surface sensitivity).^69^
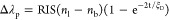
2

However, eq 2 is only valid for cases when a layer is formed on an ideally clean surface. In the experimental case reported here, two gases are exchanged, and an initial layer is replaced with the new gas, as illustrated in Figure 8. Thus, eq 2 can be extended to take into consideration the exchange of gases, both in the layer and in the bulk. Equation 3 is formulated for the exchange of two gases, where λ_p_ref__ and *n*_b_ref__ are the peak position and the bulk refractive index of the hypothetic, ideally clean state.
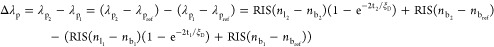
3

**Figure 9:**
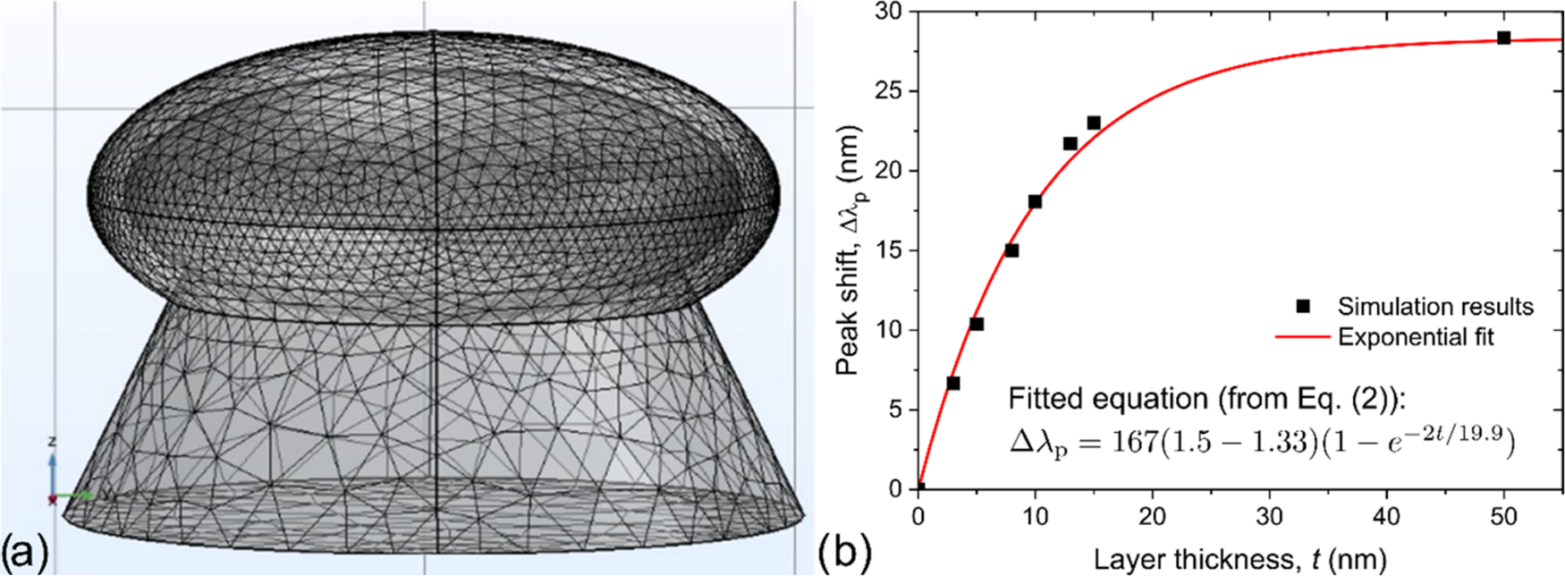
(a) The structure and mesh of the hexagonal unit cell for modeling the response of the double Au NP layer configuration. The ellipsoidal shape of the Au NP is placed on top of a SiO_2_ pillar and covered with a deposited layer of increasing thickness. For more information regarding the digital twin model see ref^70^. (b) The resulting peak shift values as a function of the deposited layer thickness was fitted using eq 2.

Note that in eq 3 the exponential relationship of eq 2 is extended with a part to incorporate the bulk RI changes upon gas exchange. However, RIS(*n*_b_2__ – *n*_b_ref__) – RIS(*n*_b_1__ – *n*_b_ref__) can be reduced to RISΔ*n*_b_, where Δ*n*_b_ represents the difference between the bulk RI of the exchanged gases. The equation can be simplified by focusing solely on the changes in a layer with an arbitrary thickness *t*, where the “effective” RI change is Δ*n*_l_, as illustrated in Figure 8.
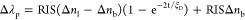
4

eq 4 is similar to the well-known eq 2, except it represents the changes in a layer with predefined thickness. In this form, eq 4 can be used to evaluate the gas sensing performance of the sensors, however, the plasmon decay length of the nanoparticle arrangements (ξ_D_) is still unknown. To determine this, digital twins (complex multiphysical models) were constructed for the three types of Au NP layers. As discussed in a previous work,^70^ the digital twin takes into account the geometry of both the ellipsoidal Au NP and the SiO_2_ pillars underneath them (see Figure 9a). The geometrical parameters in the model are fine-tuned by the four-point sensitivity calibration in different liquids, presented in Figure 5, to match the plasmon resonance peak positions. After fine-tuning the geometrical parameters, the mean square error between the experimentally measured peak positions and those resulting from the simulations is below 0.1 nm. For detailed information regarding the digital twin construction and tuning please see.^70^

To obtain ξ_D_, a layer with a fixed RI is deposited on the NP surface. By increasing the layer thickness, the plasmon field around the NP can be mapped through the peak shift and the decay length can be obtained by fitting eq 2 to the results, considering an exponential relationship. In the present case, *n*_l_ = 1.50 and *n*_b_ = 1.33 were used as inputs, which corresponds to an experimentally realizable scenario.^66^ The resulting peak shift values are presented in Figure 9b, along with the fitted exponential eq 2.

**Figure 10:**
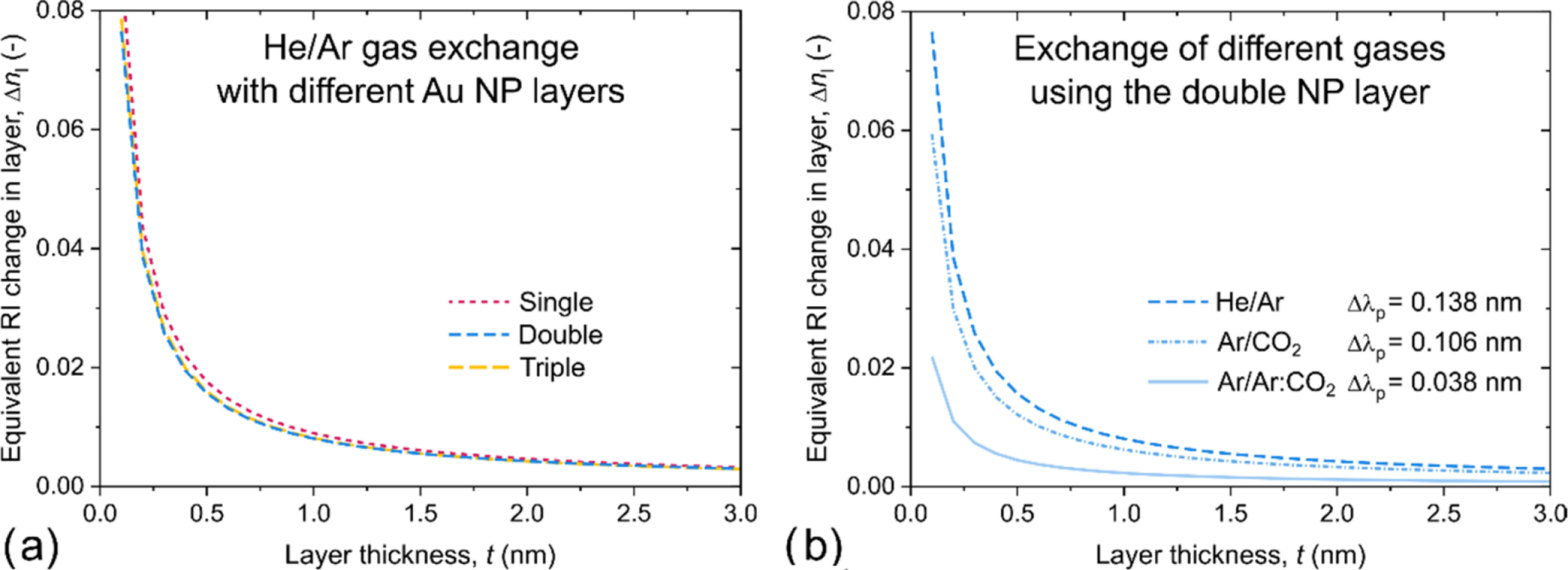
Equivalent refractive index change (Δ*n*_l_) in a gas layer of *t* for different sensing scenarios, obtained by using eq 4 on the presented experimental data. (a) He/Ar gas exchange with the three different Au NP layers. The Δλ_p_ values correspond to those presented in Figure 6. (b) Exchange of different gases using the double Au NP layer configuration. The Δλ_p_ values correspond to those presented in Figures 6 and 7.

In light of the obtained plasmon decay lengths, eq 4 can be used to evaluate the experimental data presented in Figures 6 and 7. Figure 10a compares the (Δ*n*_l_ – *t*) functions resulting from the He/Ar exchange for the three different nanoparticle arrangements (formally solving eq 4 with Δλ_p_ = λ_p_Ar__ – λ_p_He__; Δ*n*_l_ = *n*_l_Ar__ – *n*_l_He__ and Δ*n*_b_ = *n*_b_Ar__ – *n*_b_He__).

As expected, the three curves from Figure 10a have very small deviation. Although the three Au NP arrangements have different RIS and ξ_D_ values and thus their response (Δλ_p_) is also different (see Figure 6), it can be assumed that, at the same conditions (gas type and pressure), the formed layers on the Au NPs surface are the same. Thus, the Δ*n*_l_(*t*) curves are expected to overlap with each other. In Figure 10b the data obtained with the double Au NP arrangement is evaluated for different gas exchanges with eq 4. Here, the RIS and ξ_D_ values are fixed for the arrangement, but the sensor response (Δλ_p_) is different for the various gases. Thus, the Δ*n*_l_(*t*) curves change accordingly: a smaller response corresponds to a smaller (Δ*n*_l_ – *t*) pair.

### 3.5 Introducing the LSPR Gas Sensitivity Function

It was demonstrated that the proposed model can be successfully used to interpret gas sensing results in a way that is consistent with the sensitivity calibration in liquids. In most practical gas sensing scenarios, the exact thickness and refractive index of the adsorbed gas layer is not known and is influenced by many factors. Thus, using the Δ*n*_l_(*t*) functions is advised to describe the measured plasmon peak shift (Δλ_p_) upon the exchange of gases. Based on this function the proposed methodology for the benchmarking of plasmonic gas sensors’ sensitivity could be the following:

(1) Obtain the bulk refractive index sensitivity (RIS) by calibrating with media of known RI, e.g., aqueous solutions, or solvent-based refractive index standards.
(2) Obtain the gas exchange response (Δλ_p_) in accordance with the intended target application.
(3) Calculate the Δ*n*_l_(*t*) function corresponding with the sensor response by solving eq 4. For this, the decay length of the used nanoparticle or particle arrangement (ξ_D_) can either be obtained from numerical simulations or for simpler structures can be estimated based on data available in the literature.
(4) Calculate the proposed “LSPR Gas Sensitivity”, GS(*t*) benchmarking function, as defined in eq 5.

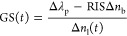
5

Similarly to the bulk refractive index sensitivity (RIS), the proposed GS(*t*) function relates the measured peak shift with the effective refractive index change, but it takes into account the inseparability of the refractive index and adsorbed layer thickness in gas sensing. GS(*t*) should be independent for the tested gases and pressures (as their influence is considered through Δ*n*_l_(*t*)) and in this form it is also compensated with the RI changes of the bulk.

**Figure 11:**
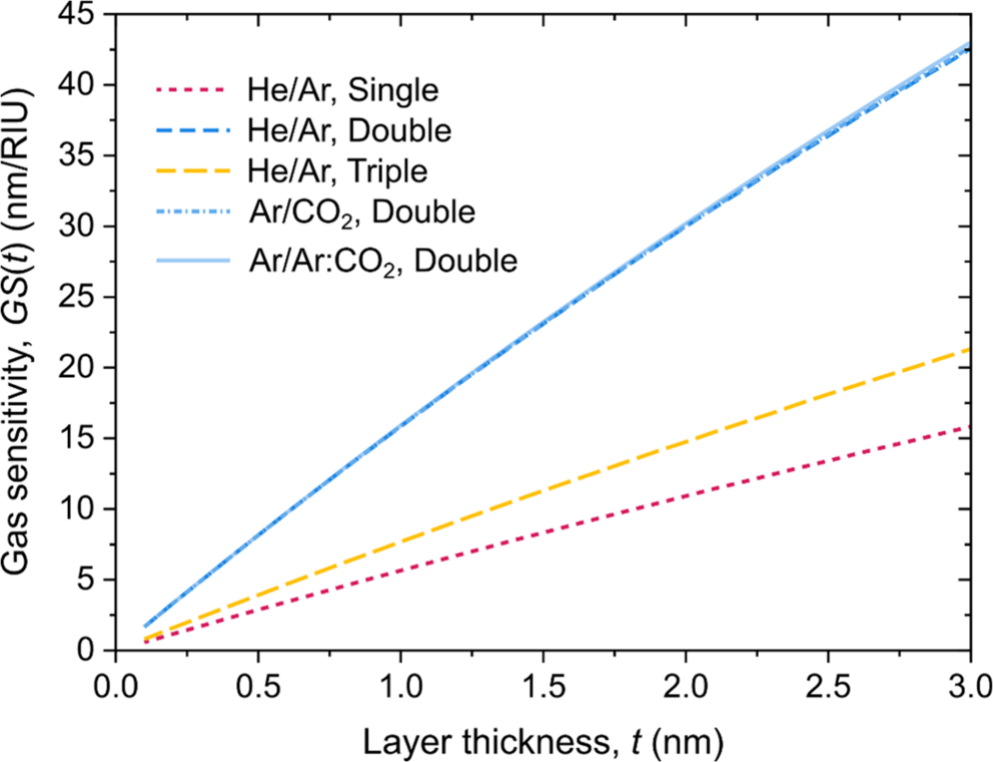
LSPR gas sensitivity functions GS(*t*) for the three tested nanoparticle arrangements calculated for different gas exchanges.

To prove these, Figure 11 presents the proposed gas sensitivity functions for the three tested nanoparticle arrangements for the different gas exchanges. As can be seen, the GS(*t*) functions are independent of the tested gas types for the double particle arrangement. The gas sensitivity functions also correlate well with the measured bulk RIS values, as expected.

Finally, one might to consider an even more practical “single parameter benchmark” to characterize the gas sensing properties of LSPR sensors. The gas sensitivity function is exponential in nature, thus can be perfectly fitted with an *y* = *a*(1 – exp(−*bx*)) function. However, for such thin layers (*t* ≤ 3 nm) a simple linear fit (*y* = *ax*) also gives a good approximation (*R*^2^ > 0.99). In other words, the derivative of the gas sensitivity function  can be used as a single parameter for benchmarking the gas sensing performance. Table 2 gives the fitted parameters of the gas sensitivity function for the three presented particle arrangements, calculated for the Ar/He exchange.

**Table 2:** Fit Parameters of the Gas Sensitivity Function for the Three Nanoparticle Arrangements

	GS(*t*) = *a*(1 – exp(−*bt*))		
arrangement type	*a*	*b*	
single	84.2	0.0694	5.14
double	148	0.113	13.6
triple	98.5	0.0812	6.89

## 4 Conclusions

Novel hexagonally ordered Au nanoparticle (NP) arrangements were fabricated and tested as highly sensitive refractive index gas sensors, using the localized surface plasmon resonance (LSPR) phenomenon to transduce a dielectric property change into a measurable optical signal. Three different Au NP arrangements (single, double and triple layers) were fabricated with oblate spheroidal shapes with increasing both diameter and height. The “intermediate” (double layer) arrangement showed the best sensing performance, either in bulk refractive index sensitivity (RIS) measurements, and gas sensing tests, reaching values in the order of 167 nm RIU^–1^ for RIS and 0.138 nm for LSPR shift response for He/Ar gas exchange. The LOD of CO_2_ detection was also investigated through Ar/CO_2_ exchange and was found to be around 10%. To interpret the gas sensing performance of the sensors, a new model was introduced that takes into account both the surface sensitivity and the plasmon decay of the nanoparticles to evaluate the measured LSPR response considering adsorbed gas layers with a Δ*n*_l_(*t*) function. Based on this model, a new benchmarking function, termed as gas sensitivity GS(*t*) was introduced. GS(*t*) characterizes the gas sensing performance of a plasmonic sensor and is independent of the type and pressure of the tested gases. It was demonstrated that the derivative of this function, , can be conveniently used as a single parameter for benchmarking purposes.

## Supporting Information Available

Calculation of nanoparticle parameters (S1) and long-term stability of the LSPR sensors (S2) (PDF)
