# Supplementary material for: New Parameter for Benchmarking Plasmonic Gas Sensors Demonstrated with Densely Packed Au Nanoparticle Layers
Source: ACS Appl Mater Interfaces. 2024 Oct 14;16(42):57832–42. doi: 10.1021/acsami.4c11102 (PMC11503611; doi:10.1021/acsami.4c11102)
Supplement: Supplementary file 1 — am4c11102_si_001.pdf [file am4c11102_si_001.pdf]

Supporting Information

New parameter for benchmarking plasmonic gas sensors demonstrated with densely-packed Au nanoparticle layers

Manuela Proença[1], Tomáš Lednický[2], Diana I. Meira[1], Marco S. Rodrigues[1], Filipe Vaz[1,3], Joel Borges[1,3], Attila Bonyár[4,5]*

[1]Physics Center of Minho and Porto Universities (CF-UM-UP), University of Minho, Campus de Azurém, 4800-058 Guimarães, Portugal

[2]Leibniz Institute of Photonic Technology, Albert-Einstein-Str. 9, 07745 Jena, Germany

[3]LaPMET - Laboratory of Physics for Materials and Emergent Technologies, University of Minho, Campus de Gualtar, 4710-057 Braga, Portugal

[4]Department of Electronics Technology, Faculty of Electrical Engineering and Informatics, Budapest University of Technology and Economics, Egry József street 18, H-1111, Budapest, Hungary

[5]Wigner Research Centre for Physics, Konkoly-Thege Miklós way 29-33, H-1121, Budapest, Hungary

Correspondence: tomas.lednicky@leibniz-ipht.de; *joelborges@fisica.uminho.pt; bonyar.attila@vik.bme.hu ;

S1. Calculation of nanoparticle parameters

The calculation process of nanoparticle (NP) parameters is present in Figure S1, as shown in the case of a double Au NP layer (SEM images). All related data, scripts, and procedures are available as dataset on Zenodo [1]. High-resolution SEM images (0.65 nm/px) were post-processed (Gwyddion 2.63 software) [2] by removing SEM artifacts originating from the substrate charging and drift correction. NPs were masked (Figure S1.b) utilizing features from Gwyddion and Adobe Photoshop 2020 software. Several images, indicated by set sizes, were processed to estimate the mean diameter of NPs as double the '*mean radius*' parameter, as shown in Table S1. The height of NPs was similarly estimated from STEM images (example in Figure S2) as double the '*Minor semiaxis of equivalent ellipse*' parameter. However, this parameter should be carefully considered, as the sample sets are significantly smaller due to the limited number of NPs in a TEM lamella. Additionally, the height measurements are highly dependent on the orientation of the NPs within the lamella and their shapes, which often deviate substantially from ideal ellipsoidal geometry. The nearest neighbor's (NN) distance parameter was also calculated using a mask from SEM images. For this, a short Matlab script was used to detect NP edges, estimate their centroids, filter centroid pairs by a threshold distance from each other (165 nm corresponding to 150% of the cell size), and calculate the shortest distance between their edges. The output of this script is visualized in Figure S1.c.

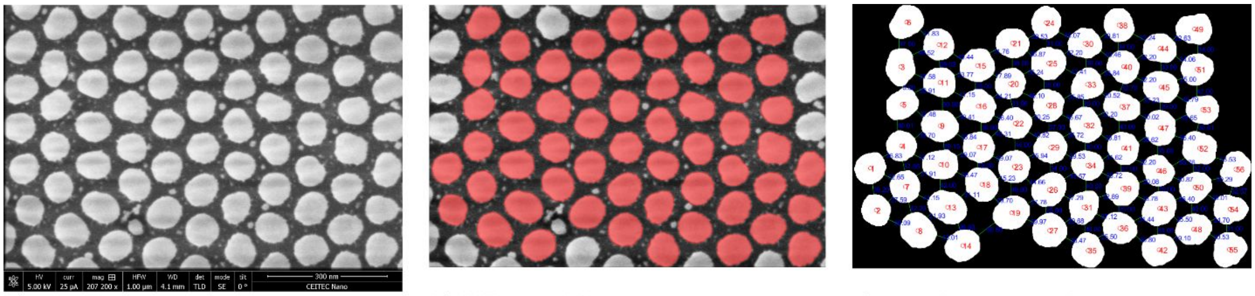

a) raw image b) NP masking c) mask processing

Figure S1. Process of NP parameters calculation from SEM images: a) raw SEM image, b) processed and masked image, and c) calculation output showing NPs' labeled centroid position with shortest distances between neighbor NPs.

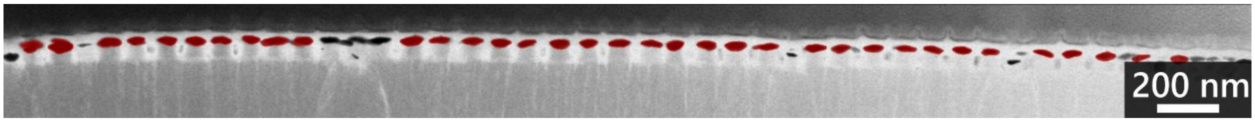

Figure S2. Masked STEM image of single Au NP layer.

Table S1. Distribution parameters of Au NPs arrangements.

Distribution Parameters	Single	Double	Triple
Avg. mean diameter (nm)	72.5 ± 4.2	81.5 ± 2.9	87.6 ± 3.8
The set size of avg. mean diameter (counts)	147	111	110
Avg. NN distance (nm)	38.5 ± 10.3	23.0 ± 5.6	15.7 ± 6.2
The set size of avg. NN distance (counts)	343	271	242
Avg. height (nm)	29.4 ± 4.8	32.8 ± 6.0	40.9 ± 4.8
The set size of avg. height (counts)	47	22	19

S2. Long-term stability of the LSPR sensors

Long-term stability was investigated in a six-month period by measuring the response of the same (double Au NP layer) sensor, as illustrated in Figure S3. The sensor was plasma cleaned (1 min, 30 W, 80 Pa) to remove hydrocarbon contamination. The degradation rate for observed period is approximately 4% decrease of the initial performance per month.

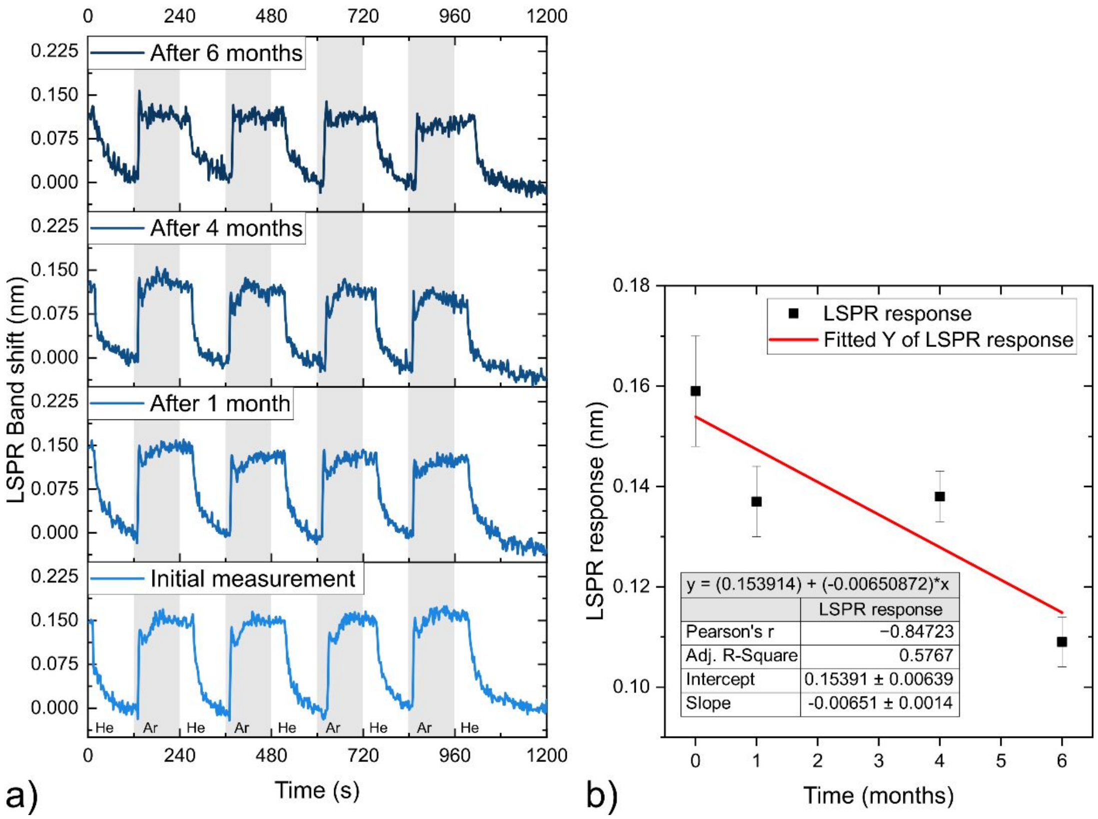

Figure S3. Double Au NP layer sensor's LSPR band wavelength response investigated in respect to the age of the sensor: a) sensor during the He vs. Ar cycles and b) LSPR response (observed total shift) decrease in the sensor life-time of 6 months.

References

1. Lednický, T. (2024). LSPR gas sensitivity: Introducing a new parameter for benchmarking plasmonic gas sensors, through a case study of densely-packed Au nanoparticle layers, Dataset on Zenodo. URL: https://doi.org/10.5281/zenodo.10783767
2. Nečas, D., Klapetek, P. Gwyddion: an open-source software for SPM data analysis. *centr.eur.j.phys.* **10**, 181–188 (2012). https://doi.org/10.2478/s11534-011-0096-2
